# WIPAKO^®^ Winterthur interprofessional training concept “communication and cooperation in health professions”: concept, development process and implementation

**DOI:** 10.3205/zma001460

**Published:** 2021-03-15

**Authors:** Brigitta Spiegel-Steinmann, Emanuel Feusi, Frank Wieber, Marion Huber

**Affiliations:** 1 ZHAW Zurich University of Applied Sciences, School of Health Professions, Institute of Health Sciences, Winterthur, Switzerland; 2 University of Konstanz, Department of Psychology, Konstanz, Germany

**Keywords:** curriculum, competency-based education, social competence, personal communication, interprofessional relations, interdisciplinary communication

## Abstract

**Objective: **The positioning and training of communicative and social competencies to improve interprofessional cooperation is and will be a challenge for educational institutions. The aim of this project was to reorient the interprofessional training elements of bachelor programs in terms of both content and structure, to improve the legally required practical relevance and to aim for a sustainable anchoring through the targeted design of the development process. WIPAKO^®^ facilitates the acquisition of communicative and social competencies as a prerequisite for cooperation between the health and medical professions.

**Methodology: **Once suitable process structures had been created, an interprofessional committee of experts defined the training elements (competencies, content, learning and teaching methods, learning objective review and evaluation) in an iterative consensus process based on various framework models and on the involvement of the various stakeholders in the individual study programs.

**Results: **A training concept focusing on communicative and social competencies for interprofessional cooperation is available. The consistent interprofessional development of the concept promoted joint responsibility for training in the sense of an organizational development process.

**Conclusion: **The outlined procedure for the conceptualization and implementation of a corresponding framework model and the framework model itself provide the basis for the empirical examination of competence acquisition and the sustainable anchoring of interprofessional training elements. This will provide suggestions for other educational institutions facing similar challenges.

## Zusammenfassung

**Zielsetzung: **Die Verortung und Ausbildung kommunikativer und sozialer Kompetenzen zur Verbesserung der interprofessionellen Zusammenarbeit fordern Bildungsinstitutionen aktuell und künftig heraus. Ziel des vorliegenden Projektes war, die interprofessionellen Ausbildungselemente der Bachelorprogramme inhaltlich wie strukturell neu auszurichten, den gesetzlich geforderten Praxisbezug zu verbessern und durch die gezielte Gestaltung des Entwicklungsprozesses die nachhaltige Verankerung anzutreben. WIPAKO^®^ ermöglicht den Erwerb kommunikativer und sozialer Kompetenzen als Voraussetzung für die Zusammenarbeit der Gesundheits- und Medizinalberufe.

**Methodik: **Nach der Schaffung geeigneter Prozessstrukturen, legte ein interprofessionell zusammengesetztes Expert*innen-Gremium in einem iterativen Konsensverfahren basierend auf verschiedenen Rahmenmodellen und dem Einbezug der verschiedenen Stakeholder der einzelnen Studiengänge die Ausbildungselemente (Kompetenzen, Inhalte, Lern- und Lehrmethoden, Lernzielüberprüfung und Evaluation) fest.

**Ergebnisse: **Ein Ausbildungskonzept mit Fokus auf kommunikative und soziale Kompetenzen zur interprofessionellen Zusammenarbeit liegt vor. Durch die konsequente interprofessionelle Erarbeitung des Konzeptes wurde im Sinne eines Organisationsentwicklungsprozesses die gemeinsame Verantwortung für die Ausbildung gefördert.

**Schlussfolgerung: **Das skizzierte Vorgehen zur Konzeption und Implementierung eines entsprechenden Rahmenmodells und das Rahmenmodell selbst schaffen Grundlagen für die empirische Überprüfung des Kompetenzerwerbes und der nachhaltigen Verankerung interprofessioneller Ausbildungselemente. Damit werden Anregungen gegeben für andere Bildungsinstitutionen, die vor ähnlichen Herausforderungen stehen.

## 1. Introduction/initial position

Both nationally and internationally, there is a demand for efficient interprofessional health care, and initial support has been established to improve it [1], [2], [3], [4], [5], [6]. The necessary skills and attitudes should be taught and acquired as early as possible in professional training [7], [8]. To this end, competencies for interprofessional and interdisciplinary cooperation must be integrated into the existing framework models of the health care and medical professions [5]. [9], [10], [11]. 

In the Anglo-American world, various framework models for training interprofessional skills [12], [13], [14], [15] have already been developed and implemented [10], [16]. In the German-speaking countries (Germany, Austria, Switzerland, the so-called DACH countries), support programs and measures are increasingly establishing themselves at the institutional and political level of the health care system that are designed to promote interprofessional education (IPE) in teaching and practice [8], [17], [18], [19]. 

Even though the training of social and communicative competencies and the integration of interprofessional teaching and learning programs in the health and medical professions in the DACH countries is generally recognized [8], [20] and the implementation of longitudinal communication curricula is well advanced [20], it is difficult to embed and consolidate IPE elements in the curriculum [21]. It seems that in addition to the (professional) political framework conditions, developments must also take place within the training organizations in order to create structures conducive to sustainable implementation [21], which is an aspect addressed in this article.

In view of these developments, and of a university strategy that aims at increasing both digitization and student numbers, it was also necessary for the School of Health Professions at Zurich University of Applied Sciences (ZHAW) to rethink and realign the goals, content, and methodological-didactic practice of IPE. 

It was decided to develop and implement a new concept along the theme Communication and Interprofessional Cooperation. A consistent, theoretical-conceptual support as well as a stronger connection to and networking with the courses of studies including their practical training were leading factors in order to anchor the IPE as an integral part of the professional training in the long term. 

The term IPE is used when two or more professions from the health and/or social care programs learn with, from and about each other in order to improve cooperation and health care [22], [23], [24]. At the ZHAW School of Health Professions, these are the bachelor's degree programs in occupational therapy (OT), health promotion and prevention (HP), midwifery (MW), nursing (NU) and physiotherapy (PT). The aim of the following article is to describe the procedure at the conceptual and process design level and to outline the content of the new concept. For better readability, we use the term 'patient' to represent patients, clients and/or client systems.

## 2. Methodology

First, the procedure regarding the design of the development process (who develops?) is described and then the concept development is discussed (how to proceed?).

### 2.1. Who develops the curricula? Process design and stakeholders

In contrast to the previous practice of having the IPE modules designed by individual persons working in IPE, all five study programs were instructed – in the sense of "turning the persons concerned into participants" – to appoint one representative each, who has proven expertise in the areas of communication and interprofessional cooperation, has strong links in his or her own study program and is well acquainted with the content of the training. 

These five experts, together with two representatives of IPT, formed a working group (N=7) with the task of developing a training concept for the thematic strand of Communication and Interprofessional Cooperation, which comprises three modules in the 2^nd^, 5^th^ and 6^th^ semesters, totaling 12 European Credit Transfer System points. Each group member had two roles: The member was to contribute his or her expertise and represent the interests of the study program and, as a multiplier, ensure coordination and coherence with the program. Based on this constellation, three simultaneous interaction processes could be distinguished:

1. Professional discussions among the representatives (expert role) served to select the relevant concepts, theories and practical contents in the field of communication and interprofessional cooperation. In most cases a consensus was reached rather quickly.
2. Intra-professional discussions with members of one’s own degree program indicate an inter-role conflict, due to contradictions between one's own expert view of the relevance of the topic and the views and needs of the stakeholders of one's own degree program, which had to be represented. Here, individual representatives of the study programs reported divergences.
3. Interprofessional discussions describe the concrete act of learning together with, from and about each other [23] at the level of the teaching staff within the framework of the task of drawing up a training concept for which they are jointly responsible. These were often led from the role of course representatives and enabled self-awareness of what interprofessional cooperation in the professional training of one's own institution could concretely mean and how this could be implemented at the level of the teaching staff.

This triple commitment to the subject matter itself, to one's own profession and to IPE formed the basis of the cooperation in the working group.

#### 2.2. How is the training concept developed? Procedures and milestones

**Figure 1:**
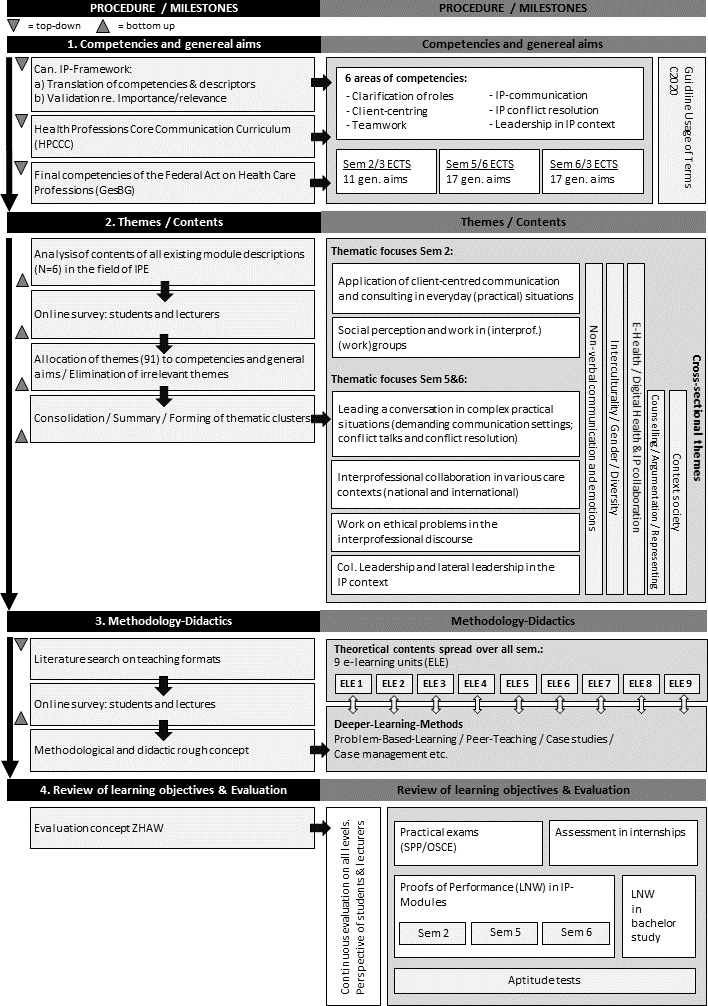
Development of the thread Communication and Interprofessional Collaboration: milestones, procedures and results. IP=interprofessional. Can=Canadian.

Based on the definition of the competencies to be achieved and the general aims, the contents were determined, the methodical-didactical implementation was defined, and the review of learning objectives and the evaluation were planned. The approach was both theory-based (top-down) and experience-based (bottom-up). The concrete procedure for the development of the training concept is illustrated in figure 1 (Fig. 1), left column, and is specified below for the various milestones. 

##### 2.2.1. Competencies and general aims

An important aspect of the competencies and general aims to be addressed was their theoretical and empirical anchoring. Starting from the Canadian framework model for the training of interprofessional competencies [13], which was translated into German by linguists, each study program evaluated the respective competencies and descriptors with regard to their importance/relevance for its own profession. In terms of face validity [25], all of them were considered important by all study programs. 

**Figure 2:**
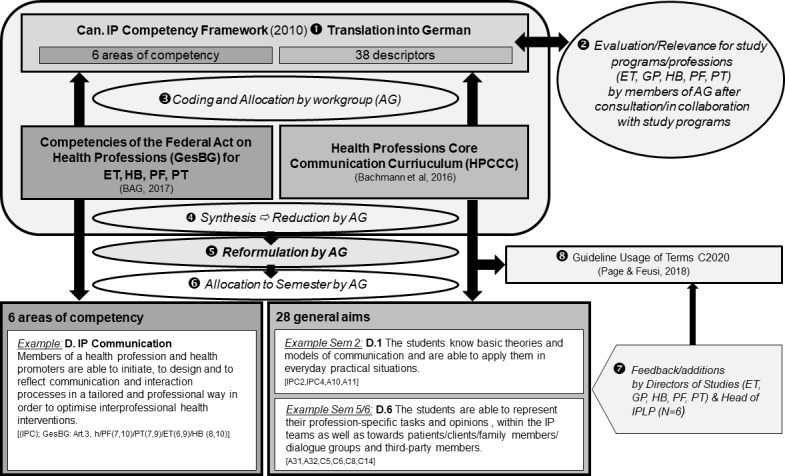
Procedure to determine the competency descriptions and the general aims. 1-8=Order of the single steps. All competencies and general aims were given indexes which refer to the corresponding contents in the frame models mentioned. ET=Occupational Therapy, GP=Health Promotion and Prevention, HB=Midwifery, PF=Nursing, PT=Physiotherapy, IPLP=Interprofessional Education and Practice.

These competencies and descriptors were compared with the German version of the Health Professions Core Communication Curriculum (HPCCC) [26] as well as with the final competencies [27] formulated in the federal act and were supplemented where necessary. The descriptions were then grouped by subject and summarized. In the interprofessional working group they were reformulated in a language understandable to all professions and were then assigned to the respective semesters (see figure 2 (Fig. 2)). 

##### 2.2.2. Themes and contents

The next step was to develop the contents. All previous module descriptions of the IPE (n=6) created by individual persons of the IPE were viewed and all themes were extracted. By means of an online survey, students of all bachelor programs and semesters (n=651/1302; response rate=50%) and all internal teachers (n=77/202; response rate=38%) were asked to assess the practical benefit in terms of face validity on a 5-point scale [25] and to add any missing topics. The resulting 91 topics were screened by the working group, assigned to competencies and general aims, condensed iteratively into thematic clusters and summarized. Topics without reference to the previously defined competencies and general aims were eliminated by consensus decision. 

##### 2.2.3. Methods and teaching formats

In the context of the online survey described above, students (n=651) and teachers (n=77) assessed various teaching formats extracted from the literature with regard to their suitability for teaching communicative and interprofessional skills on a 5-point scale, from highly effective to not effective at all. Subsequently, the results were compared with literature-based findings and the possibilities of implementing suitable teaching formats within the given personnel and financial framework were determined. 

##### 2.2.4. Review of learning objectives and evaluation

The review and evaluation of learning objectives must be taken into account from the beginning at all milestones in the development of the curriculum [28]. The aim was to implement multi-dimensional and multi-perspective reviews of learning objectives and evaluations on the basis of joint-competence descriptions and operationalizations at the places of learning – university, as well as practice.

## 3. Results

The results include both the organizational changes initiated through this process design and the resulting training concept itself.

### 3.1. Establishment of organizational structures for sustainable implementation 

**Figure 3:**
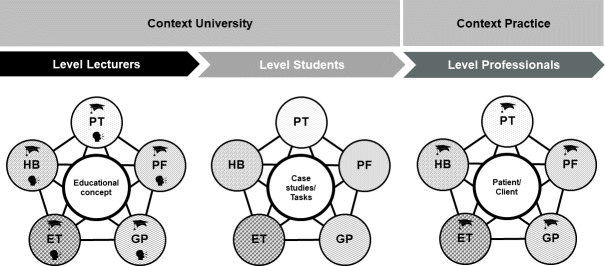
Different contexts and levels of interprofessional collaboration and cooperation. (ET=Occupational Therapy, HB=Midwifery, PT=Physiotherapy, PF=Nursing, GP=Health Promotion and Prevention).

By establishing a working group with representatives from all the study programs with the appropriate expertise, it was possible to incorporate the various perspectives of the professions on the subject matter into the development process and, at the same time, to establish a binding character of the study programs with regard to the skills and contents taught in the modules. In their role as multipliers, the members of the working group informed their study program about the milestones, obtained opinions and, thus, provoked a continuous debate on the topics of communication and inter-professional cooperation already during the conception phase. The themes became a matter of joint responsibility, which led to a more differentiated understanding of IPE and a stronger integration into the respective study programs. IPE became the trigger for interprofessional discussions, which in turn facilitated interprofessional learning at the level of those responsible for education (see figure 3 (Fig. 3)).

In analogy to the iceberg model of Schein [29], an organizational development process was initiated “in depth”, while “on the surface” the new training concept was created. 

#### 3.2. The training concept

The resulting training concept is illustrated in figure 1 (Fig. 1) (right column).

##### 3.2.1. Competencies and general aims

Following the Canadian framework model [13], six jointly reformulated areas of competence were identified, with 28 general aims, 11 for the 2^nd^ semester and 17 for the 5^th^ and 6^th^ semesters. These are now also included in the course-specific practicum assessments. It was challenging to formulate the respective competencies and general aims in such a way that all professions have the same understanding of them and identify themselves with the formulations. This process resulted in an internal guideline that regulates the use of various terms depending on which profession(s) are to be addressed [30]. 

##### 3.2.2. Themes and contents

The content assigned to the competencies and general aims was divided into thematic priorities and common principles. Thematic focuses directly address social and communicative competencies, such as the implementation of person-centered conversation within the various professions and in inter-professional exchange, as well as others. Common principles are repeatedly taken up and dealt with in a longitudinal section, but are not taught as independent subjects in the IPE, such as interculturality (see figure 2 (Fig. 2)). 

##### 3.2.3. Methods and teaching formats

**Figure 4:**
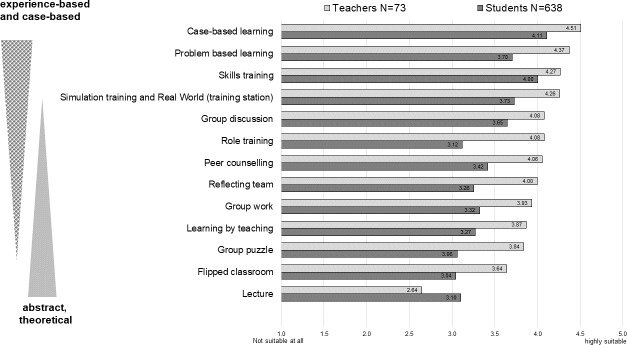
Suitability of teaching formats to acquire communicative and interprofessional competencies from the lecturers’ and students’ perspective.

Both teachers and students rated experience- and case-based teaching and learning methods as more suitable in comparison with theoretical teaching and learning formats, which confirms the methodological-didactic approaches to teaching sustainable competence acquisition discussed in the specialist literature (e.g. [31], [32]) (see figure 4 (Fig. 4)). Based on these findings, the theoretical content to be taught with learning objectives at the taxonomy level of knowledge and understanding [33], [34], [35] was prepared as an e-learning unit for self-study. These can be used independently of the interprofessional modules by all study programs and can be integrated into various teaching units. 

Contact courses, on the other hand, should be used to break down prejudices [36] and to learn with, from and about each other across professions [23]. Deeper learning methods (case work, project- and problem-oriented learning, etc.) (e.g. [37], [38], [39]) are used to design communicative and social situations in a targeted way so that interprofessional, interactive discussions can be used optimally to acquire competencies.

##### 3.2.4. Review of learning objectives and evaluation

The reviews of learning objective of the individual modules include both self-assessment and external assessment by teachers (formative, summative) and peers (formative) at the university as well as in the new learning location “practice”. They are performed by means of specific assignments and supplementary qualification requirements in the practical training assessments (see section 3.2.1.). The evaluation of the overall concept is to be based on data from module evaluations (student and teacher perspectives), graduate surveys and surveys of teachers in practical training. 

## 4. Discussion

This article describes the procedure and process design for the development of the WIPAKO^®^ training concept for communication and interprofessional cooperation and briefly introduces it. In part or in full, the concept is an integral part of the ZHAW School of Health Professions bachelor's programs.

### 4.1. Process design and actors

Reference has already been made to the need for appropriate organizational measures for sustainable development and implementation of interprofessional curricula [21]. As all study programs have been equally entrusted with the development, from the perspective of occupational and organizational psychology we can speak of shared leadership, an approach that seems to be particularly suitable for interprofessional team constellations [40], [41]. In analogy to practice, a comparable setting of interprofessional cooperation was thus created for the educational context, which led to competence enhancement at the level of the individual teachers and to further (organizational) development processes at the level of the study programs. Other authors also report that the joint-development of a curriculum strengthens the communicative, social and interprofessional skills of the participants themselves [42]. The multiplier function that the members of the working group assumed in their study programs seems to be an important moment in terms of sustainable implementation and connection to the study programs. The working group was therefore institutionalized as a specialist committee after the conception was completed, with the task and responsibility of accompanying the further design, implementation and quality of the modules, advising responsible teachers and further promoting and consolidating networking with the study programs. 

As is often the case in educational practice, the limiting factors are the limited time and personnel resources that were available for development. Expanding the working group to include trainers and students was originally intended and would have provided further valuable perspectives and discussions. 

#### 4.2. Procedures and milestones

In the absence of existing interprofessional final competencies for health care professions in Switzerland, the Canadian framework model [13] was chosen as the starting point, even if it cannot be assumed that it is directly transferable to the Swiss context [6]. The comparison with other relevant competency catalogs and the joint formulation and reformulation of the approved competencies and goals by all participating professions enabled a context-related validation [43], which should ideally be extended to other stakeholders in the future professional practice. Developing a common understanding of the competencies and goals to be addressed across the various professions seems fundamental to the curricular positioning of the various professional training programs. Nevertheless, differences in curricula and professions cannot be ruled out [44]. 

The content of the training strictly focused on the topics of communication and interprofessional cooperation. Based on the definitional contextuality of competencies [43], [45], [46], the increase in the acquisition of competencies over the three years of study is achieved through an increasing complexity of (practical) situations [43]. The first-time teaching of (competence) basics as a prerequisite for the later acquisition of interprofessional competencies (e.g., [42]) is not planned in this way, since the former is a prerequisite at the beginning of the program (part of the aptitude test). The extent to which this prioritization of content undertaken by the working group will prove its worth in practice remains open and will have to be examined in the course of the evaluation. 

The methodological-didactical strategy of designing contact courses exclusively by means of deep learning methods that provoke and promote communication and cooperation between different professions corresponds to the postulate that only then can the term interprofessional learning be used at all [47], [48] and is in-line with the view of students and teachers of the university’s own bachelor programs as well as that of Switzerland as a whole [49]. 

By establishing e-Learning units, the need for flexibility and individualization of the learning process was met [50], [51]. At the same time, these e-Learning units allow to address the heterogeneity of prior knowledge in mixed-professional student groups by varying the depth of processing. 

It is expected that a common knowledge base (Common ground, [52]) will be acquired and that this will facilitate communication within the IPE. One challenge will be to ensure the acquisition of theoretical knowledge via e-Learning unit and to optimally integrate what has been learned into classroom courses. 

In accordance with the review works by Spaulding et al. [53] and Reeves et al. [54], we expect a general trend towards the efficacy of IPE despite the heterogeneous study situation.

All in all, it can be stated that the new training concept fulfills the required theoretical anchoring and sustainable integration through participative, integrative process design [55], [56]. However, only the envisaged comprehensive concept evaluation (see section 3.4.) can provide evidence of the extent to which the new training concept and the establishment of new organizational structures have achieved the desired objectives. 

## 5. Conclusions, benefits for educational practice and further questions

The promotion of communicative and social skills to improve interprofessional cooperation poses challenges for training institutions in the health care sector that they have to face. Based on Nock's conclusions [21] that, apart from (professional) political framework conditions, measures of organizational development are also required within the training organizations, an exemplary procedure for the conception and sustainable implementation of a corresponding framework model was described here. This should provide suggestions for other educational institutions that are facing similar challenges. 

In the spirit of Confucius' statement “The way is the goal”, the authors argue that the international discourse on the content of various interprofessional framework models in the field of communication and social skills – the what – urgently needs to be expanded to include the process design and sustainable implementation of IPE – the how. Far more than a concept is needed; the development of the educational institution and the professional identity must also go hand-in-hand with it. Further theoretical-conceptual support and empirical research efforts are needed on how to design such processes. 

## Acknowledgements

An African proverb says: “If you want to go fast, go alone. If you want to go far, go together”. Thus, we would like to express our sincere thanks to the companions who helped to develop and implement this concept: Andrea Citrini (IPLP), Annette Haas (physiotherapy), Rachel Hediger (nursing), Sabine Hendriks (occupational therapy), Kerstin Jüngling (health promotion and prevention), Anita Manser (further education), Katrin Oberdörfer (midwives) (alphab.).

## Profiles

**Name of the institution: **Zurich University of Applied Sciences (ZHAW), School of Health Professions, Winterthur/Switzerland.

**Study programmes/professions: **Interprofessional training elements in the bachelor's degree programs in occupational therapy, midwifery, nursing, physiotherapy, health promotion and prevention.

**Number of students per year/semester: **Current approved enrolment as of HS2020: 546 (Occupational Therapy=90, Midwifery=90, Nursing=150, Physical Therapy=150, Health Promotion & Prevention=66). Given the ongoing shortage of skilled professionals in the health sector, further increases in student numbers are expected in the medium and long term.

**Is a longitudinal communication curriculum implemented? **from HS 2020 yes

**In which semesters are communicative and social competences taught?** Interprofessional education elements are located in the 2^nd^, 5^th^ and 6^th^ semesters (complementary to elements in the degree programs).

**Which teaching formats are used?**

- case-based learning (case discussions, etc.)
- problem-based learning (currently not, in the new 2020 curriculum)
- skills training (interview, role plays etc.)
- simulation training
- group discussions/group reflections
- various reflection formats (written, oral, etc.)
- collegial consultation
- lectures/frontal teaching => in 2020 mostly replaced by e-learning units

**In which semesters are communicative and social competences tested (formatively or pass/fail and/or with marks)? **2^nd^, 5^th^ and 6^th^ semester as well as in the practical training (internships)

**Which exam formats are used?**

- multiple choice exams
- case work with role plays / film
- examination discussions
- written papers (individual and group work)
- individual and group examinations (oral) with presentations
- behavioral observations and assessments / feedback in the context of practical training (internships)

**Who (e.g. clinic, institution) is in charge of development and implementation?** ZHAW, School of Health Professions and persons responsible for this in the individual Bachelor's degree programs, including Interprofessional Teaching and Practice (IPLP). The development also involved the stakeholder groups practice (clinics, institutions) and students of the various bachelor's degree programs.

## Current professional roles of the authors

- Brigitta Spiegel-Steinmann: Brigitta Spiegel-Steinmann heads the Division of Communication and Interprofessional Cooperation at the Interprofessional Education and Collaborative Practice Unit (IPECP) at the ZHAW School of Health Professions. She leads the development and implementation of all interprofessional modules in this subject area and is doing her doctorate at the University of Konstanz on "Interprofessional Competencies in the Health and Medical Professions".
- Emanuel Feusi: Emanuel Feusi, head of the Interprofessional Education and Collaborative Practice Unit (IPECP) and lecturer at the ZHAW School of Health Professions, deals with and teaches interprofessional learning, teaching and collaborative practice. He is responsible for the multi- and interprofessional educational elements in the Bachelor Degree Programmes and ensures their implementation.
- Prof. Dr. Frank Wieber: Frank Wieber is deputy head of the Health Sciences Research Centre at the ZHAW School of Health Professions and lecturer in the Department of Psychology at the University of Konstanz. His research and teaching focuses on the promotion of mental health in childhood and adolescence, strategies for sustainable behavioral change and interprofessionalism in the healthcare system.
- Prof. Dr. Marion Huber: Marion Huber is deputy head of the Interprofessional Education and Collaborative Practice Unit (IPECP) at the ZHAW School of Health Professions and leads the Interprofessionalism research group. Her research and teaching focuses the topic of interprofessionalism in the healthcare system, in particular the interprofessional competences development in education, practice and teaching. Teaching and project evaluations from an interprofessional perspective are part of this focus.

## Competing interests

The authors declare that they have no competing interests.
